# Evaluation of multiple-vendor AI autocontouring solutions

**DOI:** 10.1186/s13014-024-02451-4

**Published:** 2024-05-31

**Authors:** Lee Goddard, Christian Velten, Justin Tang, Karin A. Skalina, Robert Boyd, William Martin, Amar Basavatia, Madhur Garg, Wolfgang A. Tomé

**Affiliations:** 1 Department of Radiation Oncology, Montefiore Medical Center, Bronx, NY 10467 USA; 2 Albert Einstein College of Medicine, Bronx, NY 10461 USA; 3 Division of Medical Physics, Albert Einstein College of Medicine, 1300 Morris Park Ave, Block Building Room 106, Bronx, NY 10461 USA

**Keywords:** AI, Autocontouring

## Abstract

**Background:**

Multiple artificial intelligence (AI)-based autocontouring solutions have become available, each promising high accuracy and time savings compared with manual contouring. Before implementing AI-driven autocontouring into clinical practice, three commercially available CT-based solutions were evaluated.

**Materials and methods:**

The following solutions were evaluated in this work: MIM-ProtégéAI+ (MIM), Radformation-AutoContour (RAD), and Siemens-DirectORGANS (SIE). Sixteen organs were identified that could be contoured by all solutions. For each organ, ten patients that had manually generated contours approved by the treating physician (AP) were identified, totaling forty-seven different patients. CT scans in the supine position were acquired using a Siemens-SOMATOMgo 64-slice helical scanner and used to generate autocontours. Physician scoring of contour accuracy was performed by at least three physicians using a five-point Likert scale. Dice similarity coefficient (DSC), Hausdorff distance (HD) and mean distance to agreement (MDA) were calculated comparing AI contours to “ground truth” AP contours.

**Results:**

The average physician score ranged from 1.00, indicating that all physicians reviewed the contour as clinically acceptable with no modifications necessary, to 3.70, indicating changes are required and that the time taken to modify the structures would likely take as long or longer than manually generating the contour. When averaged across all sixteen structures, the AP contours had a physician score of 2.02, MIM 2.07, RAD 1.96 and SIE 1.99. DSC ranged from 0.37 to 0.98, with 41/48 (85.4%) contours having an average DSC ≥ 0.7. Average HD ranged from 2.9 to 43.3 mm. Average MDA ranged from 0.6 to 26.1 mm.

**Conclusions:**

The results of our comparison demonstrate that each vendor’s AI contouring solution exhibited capabilities similar to those of manual contouring. There were a small number of cases where unusual anatomy led to poor scores with one or more of the solutions. The consistency and comparable performance of all three vendors’ solutions suggest that radiation oncology centers can confidently choose any of the evaluated solutions based on individual preferences, resource availability, and compatibility with their existing clinical workflows. Although AI-based contouring may result in high-quality contours for the majority of patients, a minority of patients require manual contouring and more in-depth physician review.

## Background

Delineations of organs-at-risk (OARs) is a critical task in radiation therapy to define and outline the normal tissue whose radiation dose tolerances must be observed to limit associated treatment-related toxicities. Various members of the radiation oncology care team, mostly dosimetrists and radiation oncologists, are involved in creating these normal tissue delineations; however, medical physicists and/or radiation therapists may also be involved. These delineations are performed manually using various tools, such as freehand drawing tools, Hounsfield unit (HU) thresholding, Boolean operations, etc. This manual contouring is often a time-consuming and subjective process, as different individuals may contour the same structure differently [1–3]. While profound efforts have been made to create contouring guidelines to help reduce this variability [4], OAR and target contouring variability remain among the largest sources of uncertainty in the radiation therapy process [5, 6]. To reduce inter-observer variability [7], improve workflow efficiency [8] and provide necessary tools for adaptive radiation therapy [9, 10], automated contouring tools leveraging a variety of technologies have been developed by several vendors.

Atlas-based tools utilized predefined anatomical templates (atlases) created using manual contouring techniques to assist in OAR delineation [11]. A library of atlas patients is created that ideally represents the patient population. When an atlas is selected to generate contours, the new patient is matched to the most similar patient from the library (source) and deformably registered to the patient (target). OAR contours are then transferred to the target patient using this deformable registration. Although these atlas-based tools reduce contouring time, studies have shown that there is still substantial variability in the generated contours [7]. As atlas tools rely on a finite number of cases, they are of limited use in complex or nonstandard anatomies or in patient populations that were not included in the atlas data [12].

In recent years, multiple artificial intelligence (AI)-based autocontouring software solutions have become widely available. Compared with previous autocontouring software, AI-based solutions have been shown to have improved accuracy, reduced time requirements and fewer modifications of the generated structures required [13–16]. AI contouring tools use complex algorithms and machine learning techniques to automatically generate OAR contours. Although these contours can be more accurate than atlas-based contours, manual adjustment may still be necessary. Contouring adjustment may be performed by a dosimetrist, or other appropriately trained staff, however, final review and approval by a radiation oncologist is required. Manual adjustment of automatically generated contours can be time consuming to the point of no time savings over manual contouring [17, 18], depending on the individual user’s skill level.

Before implementing AI-driven autocontouring in our clinical practice, three commercially available CT-based AI autocontouring solutions were evaluated through physician scoring and similarity metrics to verify their accuracy and determine which, if any, of the available solutions would have sufficient accuracy to be clinically applicable. Each of these solutions utilizes individual deep learning AI-based algorithms to generate between thirty-seven and eighty-five organ contours, based on the acquired CT imaging data. Sixteen organs that could be contoured by all three of the available solutions across a range of anatomical sites including head and neck, thorax, abdomen, and pelvis were investigated. Differences in training data, neural network architecture and contour definitions, such as the superior border of the heart, lead to differences between the individual solutions and hence, the need for evaluation before clinical implementation. While this paper focuses on comparing AI autocontouring solutions, providing detailed descriptions of each algorithm is beyond its scope. Interested readers are encouraged to refer to the respective white papers cited for in-depth information on each algorithm’s specifics [19–21].

## Methods

In this study, three autocontouring solutions were compared to manual contouring. The three solutions used were ProtégéAI + v7.2.7, MIM Software Inc. (Beachwood, OH, USA) (MIM), AutoContour v2.2.8 RADformation Inc. (New York, NY, USA) (RAD), DirectORGANS v.a.40 S Healthineers (Erlangen, Germany) (SIE). Sixteen organs that were common to all three were identified: bladder, brain, brainstem, esophagus, eyes, femoral heads, heart, kidneys, liver, lungs, mandible, oral cavity, parotids, rectum, submandibular glands, and spinal cord. For each of these organs, ten patients who had manually generated contours approved by the treating physician (AP) were identified. For bilateral organs, five patients were utilized, with the left and right organs contoured individually. For the spinal cord, five patients had thoracic scans, and five had abdominal/pelvic scans, ensuring that the entire spinal cord was investigated. AP contours were approved by one of eight attending physicians at our institution with between five and twenty-eight years of experience. A total of forty-six patients were included: seventeen females (37%) and twenty-nine males (63%). All patients were simulated in a supine position on a Siemens SOMATOM go 64-slice helical CT scanner. Each scan was reconstructed with either a 1–2 mm slice thickness, depending on the intended treatment technique. These CT scans were exported to each of the three contouring solutions and contour sets generated. Images and contours were anonymized, randomized, and reviewed by at least three physicians who were blinded to the contouring technique used to generate a given contour set. Reviewing physicians had at least three years of experience in Radiation Oncology and a maximum of twenty-eight years of experience.

A five-point Likert scale (1: use as-is; 5: unusable), modified from a scale previously utilized for the review of automated RT plans [22], was utilized for physician review. A score of 1 indicates that the structures are clinically acceptable and can be used for treatment without change. A score of 2 indicates minor edits that are not necessary and that stylistic changes may occur; however, these edits are not clinically important, and current structures are clinically acceptable. A score of 3 indicates minor edits that are necessary and can be made in less time than starting from scratch or are expected to have minimal effect on treatment outcome. A score of 4 indicates major edits that are necessary and are sufficiently significant that the user would prefer to start from scratch. Finally, a score of 5 indicates the quality of the automatically generated structures is so poor that they are unusable, incorrect structures may be contoured, or no structure may be generated.

The Dice similarity coefficient (DSC) [23], Hausdorff distance (HD) [24], and mean distance to agreement (MDA) [25] were calculated for each structure using the AP contour as the ground truth. To calculate these similarity metrics, all structures were transferred to the same CT image, and contour statistics were analyzed in MIM. As AI contouring solutions typically contour the esophagus and spinal cord over the whole organ length or the length of the CT vs. the physician contours, which are mostly restricted to the area of the PTV, the AI contours were modified to include only the length of the physician contours to allow unbiased review and volumetric comparisons.

## Results

**Fig. 1:**
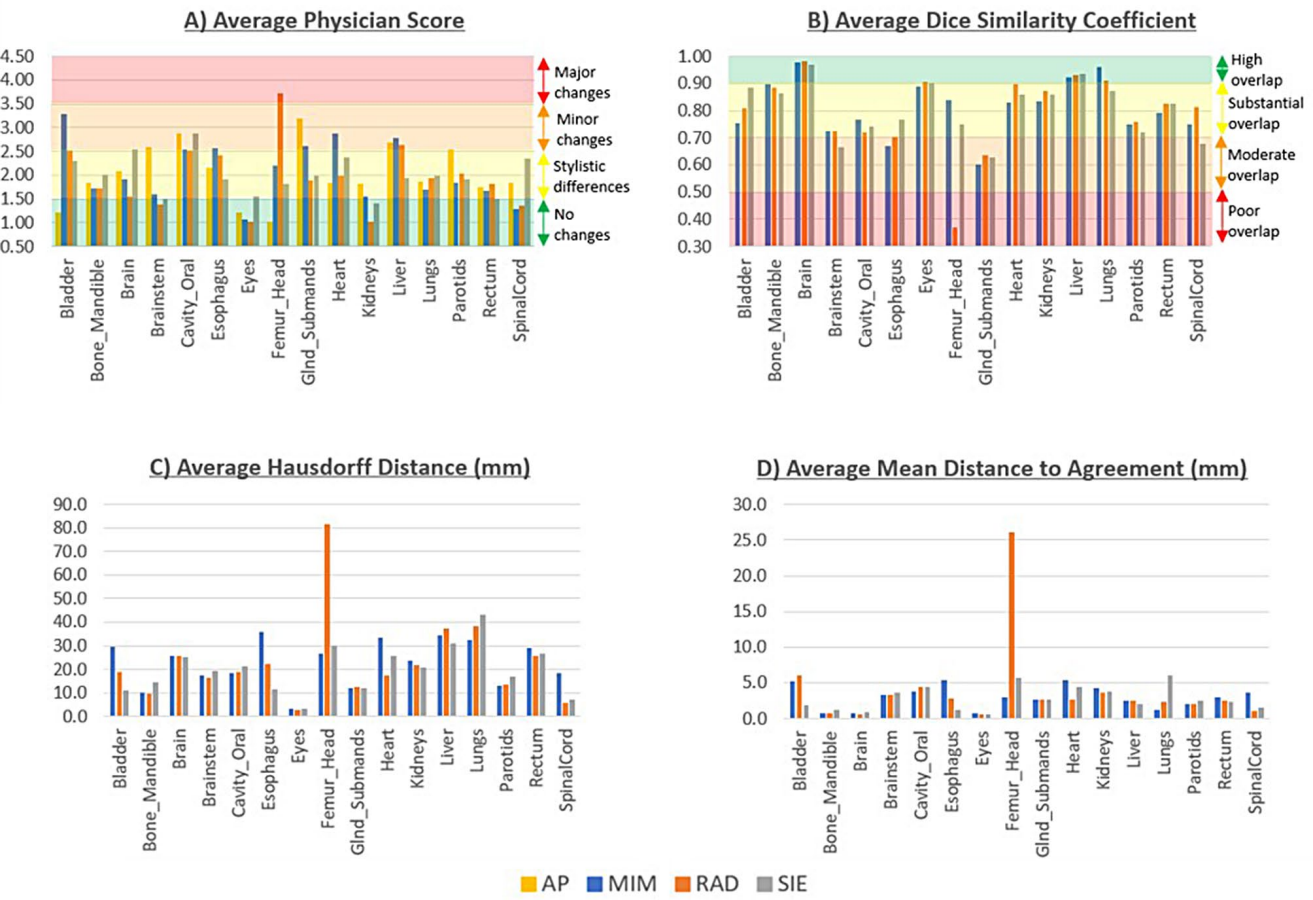
**A**) Average physician score, **B**) average Dice similarity coefficient, **C**) average Hausdorff distance (mm) and **D**) average mean distance to agreement (mm). Contours are labeled as approved by physicians (AP), generated using ProtégéAI+ (MIM), AutoContour (RAD), and DirectORGANS (SIE). Larger than typical values are shown for the femoral head due to RAD contouring the femoral head where PA, MIM and SIE also include the femoral neck

Physician scoring results are shown in Table 1 and Fig. 1A as averages and sample standard deviations for each contour. The average scores ranged from 1.00, indicating that all physicians reviewed the contour as clinically acceptable, with no modifications necessary or stylistic differences found, to 3.70, indicating that changes are required and that the time taken to modify the structures would likely take as long or longer than manually generating the contour. Overall, 12/64 (18.8%) contours had average scores ≤ 1.5, 37/64 (57.7%) had scores > 1.5 but ≤ 2.5, 12/64 (18.8%) had scores > 2.5 but ≤ 3.5 and 3/64 (4.7%) had scores > 3.5. The AP contours had an overall average of 2.02, MIM had an average of 2.07, RAD had an average of 1.96 and SIE had an average of 1.99.

**Table 1:** Average Physician Score with standard deviations of scores for manually generated approved physician contours (AP) and autocontours from MIM Software Inc. (MIM), RADformation Inc. (RAD) and Siemens Healthineers (SIE). One-way ANOVA F and *P* values are also shown. Minimum values and statistically significant *P* values are shown in **bold** *Left or right

Organ	AP	MIM	RAD	SIE	F value	*P* value
Bladder	**1.20** (0.23)	3.27 (1.24)	2.50 (1.43)	2.30 (1.41)	5.178	**0.004**
Bone_Mandible	1.83 (0.76)	**1.70** (0.51)	**1.70** (0.43)	2.00 (0.57)	0.607	0.61
Brain	2.07 (0.91)	1.90 (0.45)	**1.53** (0.23)	2.53 (0.63)	4.628	**0.008**
Brainstem	2.57 (1.10)	1.60 (0.44)	**1.37** (0.19)	1.50 (0.42)	7.403	**< 0.001**
Cavity_Oral	2.87 (0.95)	2.53 (0.71)	**2.50** (0.67)	2.87 (0.88)	0.628	0.60
Esophagus	2.15 (0.69)	2.56 (0.75)	2.41 (0.67)	**1.91** (0.41)	1.999	0.13
Eye*	1.20 (0.24)	1.07 (0.16)	**1.00** (0.00)	1.53 (0.74)	5.644	**0.003**
Femur_Head*	**1.00** (0.00)	2.20 (0.67)	3.70 (0.71)	1.80 (1.69)	13.50	**< 0.001**
Glnd_Submand*	3.19 (1.53)	2.59 (0.81)	**1.89** (0.24)	1.96 (0.20)	5.118	**0.005**
Heart	**1.83** (0.45)	2.87 (0.78)	1.97 (0.36)	2.35 (0.92)	4.847	**0.006**
Kidney*	1.80 (0.86)	1.53 (0.80)	**1.00** (0.00)	1.40 (0.31)	2.990	**0.04**
Liver	2.67 (0.70)	2.77 (1.01)	2.63 (0.99)	**1.93** (0.97)	1.709	0.18
Lung*	1.85 (0.53)	**1.69** (0.29)	1.92 (0.91)	1.98 (0.97)	0.297	0.83
Parotid*	2.53 (0.86)	**1.83** (0.28)	2.03 (0.81)	1.90 (0.82)	1.868	0.15
Rectum	1.73 (0.82)	1.67 (0.42)	1.80 (0.65)	**1.50** (0.18)	0.584	0.63
SpinalCord	1.84 (0.57)	**1.29** (0.28)	1.35 (0.35)	2.33 (0.69)	9.495	**< 0.001**

Table 2 and Fig. 1B show the average and sample standard deviation of the DSC data. The DSC ranged from 0.37 (RAD: femur-head) to 0.98 (RAD: brain). Only 1/48 (2%) contours have an average DSC < 0.5, indicating poor overlap and significant differences between the segmented region and the ground truth. A total of 6/48 (12.5%) had average DSC values between 0.5 and 0.7, indicating moderate overlap with some agreement; however, AI segmentation may still need improvement. A total of 32/48 (66.7%) had an average DSC between 0.7 and 0.9, indicating substantial overlap and showing good agreement between the AI contour and the ground truth AP contour. A total of 9/48 (18.8%) have average DSC values ≥ 0.9; these contours have a high overlap, and there is excellent agreement between the contours. When the DSCs for all structures are averaged, MIM and SIE both have averages of 0.81, while RAD has an average of 0.80.

**Table 2:** Average Dice Similarity Coefficient with standard deviations for autocontours from MIM Software Inc. (MIM), RADformation Inc. (RAD) and Siemens Healthineers (SIE). One-way ANOVA F and *P* values are also shown. Minimum values and statistically significant *P* values are shown in bold *Left or right

Organ	MIM	RAD	SIE	F value	*P* value
Bladder	**0.76** (0.24)	0.81 (0.31)	0.89 (0.09)	0.814	0.45
Bone_Mandible	0.90 (0.07)	0.89 (0.06)	**0.86** (0.10)	0.557	0.58
Brain	0.98 (0.01)	0.98 (0.01)	**0.97** (0.01)	2.502	0.10
Brainstem	0.72 (0.19)	0.73 (0.21)	**0.67** (0.19)	0.302	0.74
Cavity_Oral	0.77 (0.10)	**0.72** (0.14)	0.74 (0.14)	0.328	0.72
Esophagus	**0.67** (0.14)	0.70 (0.11)	0.77 (0.10)	1.711	0.20
Eye*	**0.89** (0.04)	0.90 (0.03)	0.90 (0.03)	0.269	0.77
Femur_Head*	0.84 (0.13)	**0.37** (0.20)	0.75 (0.34)	10.95	**< 0.001**
Glnd_Submand*	**0.60** (0.17)	0.64 (0.19)	0.63 (0.22)	0.088	0.92
Heart	**0.83** (0.14)	0.90 (0.06)	0.86 (0.16)	0.685	0.51
Kidney*	**0.83** (0.22)	0.87 (0.22)	0.86 (0.22)	0.081	0.92
Liver	**0.92** (0.03)	0.93 (0.02)	0.94 (0.02)	0.566	0.57
Lung*	0.96 (0.01)	0.91 (0.19)	**0.87** (0.30)	0.479	0.62
Parotid*	0.75 (0.10)	0.76 (0.10)	**0.72** (0.12)	0.381	0.69
Rectum	**0.79** (0.11)	0.82 (0.07)	0.83 (0.08)	0.468	0.63
SpinalCord	0.75 (0.24)	0.81 (0.11)	**0.68** (0.10)	1.727	0.20

Table 3 and Fig. 1C show the average and standard deviation of the HD. The average HD ranged from 2.9 mm (RAD: eye) to 43.3 mm (SIE: lung). Overall, 24/48 (50.0%) had an average HD > 20.0 mm. A total of 17/48 (35.4%) had an average HD between 10.0 mm and 20.0 mm. A total of 4/48 (8.3%) had an average HD between 5.0 mm and 10.0 mm, and 3/48 (6.3%) had an average HD < 5 mm. When the HD for all structures are averaged MIM had an average of 22.7 mm, RAD had an average of 23.1 mm, and SIE had an average of 20.0 mm.

**Table 3:** Average Hausdorff Distance with standard deviations (mm) for autocontours from MIM Software Inc. (MIM), RADformation Inc. (RAD) and Siemens Healthineers (SIE). One-way ANOVA F and *P* values are also shown. Minimum values and statistically significant *P* values are shown in **bold** *Left or right

Organ	MIM	RAD	SIE	F value	*P* value
Bladder	29.4 (30.0)	18.8 (25.3)	**11.1** (10.9)	1.539	0.23
Bone_Mandible	9.9 (8.1)	**9.8** (7.3)	14.5 (12.2)	0.795	0.46
Brain	25.5 (12.9)	25.4 (13.2)	**25.0** (8.6)	0.004	1.00
Brainstem	17.4 (13.8)	**16.3** (14.3)	19.4 (10.0)	0.151	0.86
Cavity_Oral	**18.5** (7.1)	18.9 (7.6)	21.4 (10.1)	0.344	0.71
Esophagus	35.9 (43.2)	22.5 (26.7)	**11.6** (7.6)	1.689	0.20
Eye*	3.3 (0.9)	**2.9** (1.0)	3.5 (2.1)	1.036	0.37
Femur_Head*	**26.6** (26.6)	81.5 (19.9)	30.0 (30.2)	14.13	**< 0.001**
Glnd_Submand*	12.3 (4.6)	12.5 (5.4)	**11.9** (5.7)	0.035	0.97
Heart	33.5 (24.2)	**17.3** (7.0)	25.8 (28.0)	1.375	0.27
Kidney*	23.6 (26.9)	21.8 (25.5)	**20.9** (25.1)	0.029	0.97
Liver	34.4 (16.4)	37.3 (27.7)	**31.0** (17.6)	0.216	0.81
Lung*	**32.5** (16.7)	38.4 (18.5)	43.3 (28.7)	0.613	0.55
Parotid*	**13.1** (3.4)	13.6 (5.8)	17.1 (7.8)	1.312	0.29
Rectum	29.3 (12.0)	**25.7** (12.7)	26.4 (15.9)	0.241	0.79
SpinalCord	18.3 (23.3)	**6.0** (2.2)	7.2 (6.2)	2.376	0.11

Table 4 and Fig. 1D show the average and standard deviation of the MDA. The average MDA ranged from 0.6 mm (RAD: eye) to 26.1 mm (RAD: femoral head). A total of 1/48 (2.1%) had an average MDA > 10.0 mm. A total of 6/48 (12.5%) had an average MDA between 5.0 mm and 10.0 mm. 21/48 (43.8%) of the MDA values were between 2.5 mm and 5.0 mm, and 20/48 (41.7%) had an average MDA < 2.5 mm. When the MDA for all the structures are averaged MIM had an average of 3.0 mm, RAD had an average of 4.0 mm, and SIE had an average of 2.8 mm.

**Table 4:** Average Mean Distance to Agreement with standard deviations (mm) for autocontours from MIM Software Inc. (MIM), RADformation Inc. (RAD) and Siemens Healthineers (SIE). One-way ANOVA F and *P* values are also shown. Minimum values and statistically significant *P* values are shown in **bold** *Left or right

Organ	MIM	RAD	SIE	F value	*P* value
Bladder	5.3 (6.5)	6.0 (14.4)	**1.8** (1.9)	0.588	0.56
Bone_Mandible	**0.7** (0.8)	0.8 (0.6)	1.1 (1.5)	0.514	0.60
Brain	0.8 (0.2)	**0.7** (0.3)	0.9 (0.3)	1.632	0.21
Brainstem	**3.2** (3.5)	3.3 (3.8)	3.6 (2.9)	0.027	0.97
Cavity_Oral	**3.8** (2.1)	4.4 (2.5)	4.4 (2.6)	0.200	0.82
Esophagus	5.5 (6.9)	2.8 (4.2)	**1.2** (0.6)	2.087	0.14
Eye*	0.7 (0.3)	**0.6** (0.2)	0.7 (0.2)	0.274	0.76
Femur_Head*	**3.0** (3.9)	26.1 (16.4)	5.8 (9.7)	12.65	**< 0.001**
Glnd_Submand*	2.7 (1.5)	**2.6** (1.8)	2.7 (1.9)	0.005	1.00
Heart	5.4 (5.8)	**2.7** (1.3)	4.5 (6.7)	0.729	0.49
Kidney*	4.3 (8.7)	**3.6** (8.2)	3.8 (8.1)	0.017	0.98
Liver	2.5 (1.3)	2.5 (1.6)	**2.1** (0.9)	0.435	0.65
Lung*	**1.2** (0.4)	2.4 (4.2)	6.0 (15.5)	0.743	0.49
Parotid*	2.0 (0.8)	**2.0** (0.8)	2.5 (1.0)	0.941	0.40
Rectum	3.0 (1.7)	2.5 (1.2)	**2.4** (1.4)	0.587	0.56
SpinalCord	3.6 (7.1)	**1.0** (0.6)	1.6 (0.7)	1.057	0.36

## Discussion

All four investigated contouring solutions obtained comparable physician scores. However, there were notable exceptions for the three AI contouring solutions for the bladder, brain, femoral head, and spinal cord, as discussed in detail below.

**Fig. 2:**
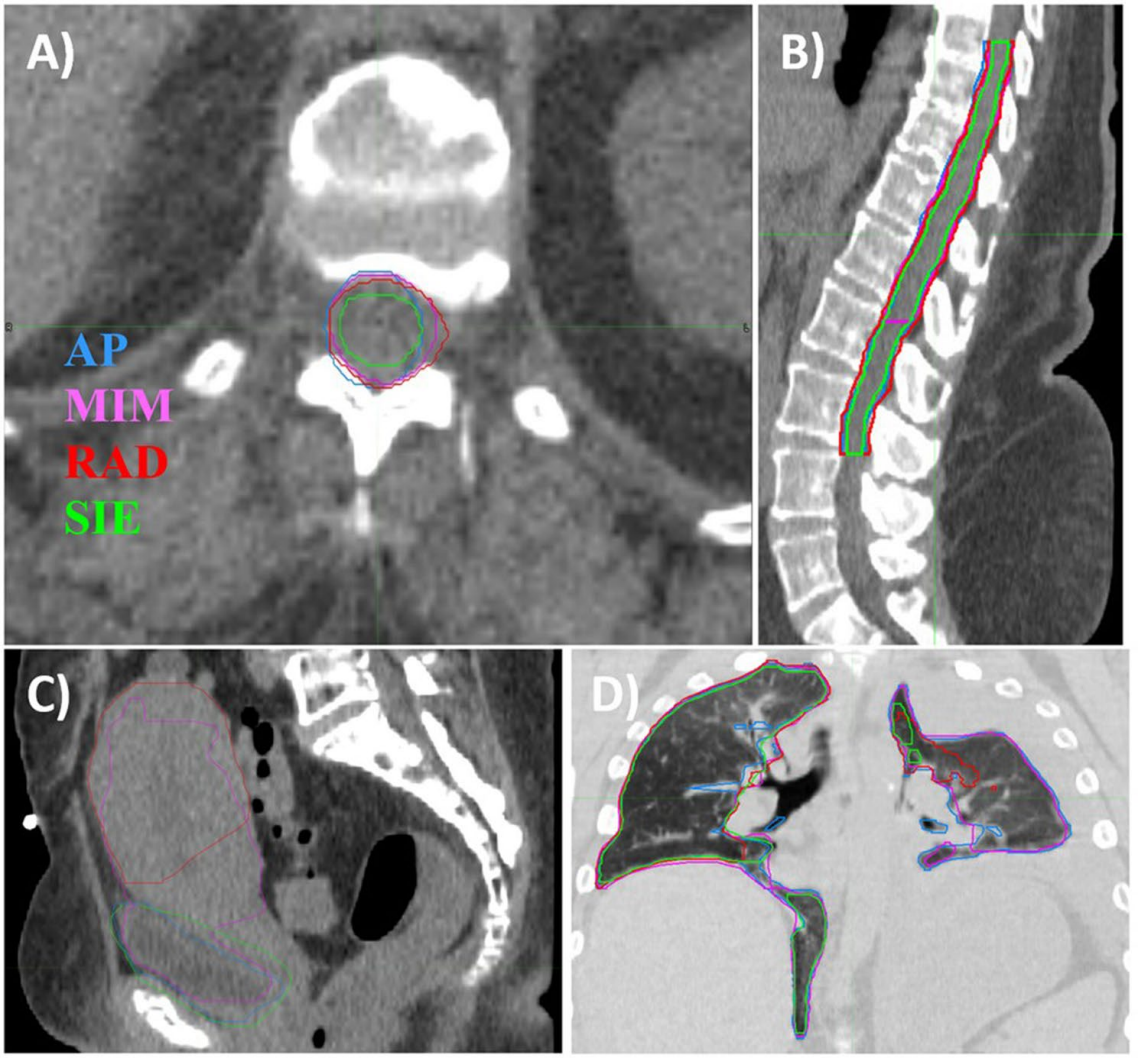
**A**) Transverse and **B**) sagittal views with a “soft tissue” window/level, showing spinal cord contours. **C**) Sagittal view with a “soft tissue” window/level, showing bladder contours. **D**) Coronal view with a “lung” window/level showing left and right lung contours. Contours are labeled as approved by physicians (AP), generated using ProtégéAI+ (MIM), AutoContour (RAD), and DirectORGANS (SIE)

Although SIE scored slightly higher (worse) physician scores than MIM or RAD for the brain contours, this can be explained by stylistic differences: SIE subtracts the brainstem from the brain contour, which is not consistent with our clinical practice and hence has higher (worse) physician scores. Similarly, for the femoral head contours, RAD contours just the femoral head and do not include the neck of the femoral head, which is included in the physician contours, MIM and SIE. Finally, for the spinal cord, SIE contours the supposed true spinal cord, whereas the AP, MIM and RAD contour the spinal canal (or thecal sac) as a surrogate for the cord, which is in concordance with our clinical practice, as shown in Fig. 2. For the spinal cord contour, while similar DSCs were found (0.75 - MIM, 0.81 - RAD, 0.68 - SIE), MIM showed a larger average HD (18.3 mm - MIM, 6.0 mm - RAD, 7.2 mm - SIE) and MDA (3.6 mm - MIM, 1.0 mm - RAD, 1.6 mm - SIE). Closer investigation revealed that these larger distances were found only in abdominal/pelvic patients where MIM contoured the spinal cord to the level of the L2 vertebra, whereas the physician and other contouring solutions included the cauda equina in the spinal cord structure, as shown in Fig. 2B.

When examining the bladder contours, poor scores were found for some or all vendors when unusual anatomy was encountered. MIM, RAD and SIE all received average scores > 4.5 for one patient where a contrast agent was placed within the bladder. Both MIM and RAD had average scores > 4.5, or “unusable”, for one female patient with advanced gynecological cancer for whom SIE had an average score of 2.67, as shown in Fig. 2C. One male patient with metastatic prostate disease and an enlarged, trabeculated bladder also received average scores > 4.5 for MIM and SIE, whereas RAD received an average score of 3.00. When these three examples of unusual anatomy were excluded, the average physician scores improved by 0.70, 0.79 and 0.73 for MIM, RAD and SIE, respectively.

An example of potential errors introduced by autocontouring solutions for patients with abnormal or nonstandard anatomy is shown in Fig. 2D. Here, the patient’s right lung was typical, while the left lung had partially collapsed. For the right lung, all autocontouring solutions performed well, with PS values between 1.67 and 2.56, DSC values ≥ 0.92 and MDA values ≤ 2.4 mm. For the left lung, however, only MIM matches the AP contour well, with a PS of 2.33, DSC of 0.93 and MDA of 1.1 mm, while both RAD and SIE produce unusable contours with DSCs of 0.38 and 0.02, respectively, and PS > 4.

These examples highlight some of the challenges faced by vendors as contouring atlases used in the definitions of specific organs may vary between research studies, internationally and over time, which can lead to the stylistic difference noted. Collaboration with users at a range of clinical practices is important to allow for improvements in these autocontouring solutions. Since we began this evaluation there have already been updates to the available models from Radformation that allow for users to select femoral head models that match the RTOG guidelines, which would theoretically improve the physician scores for this structure. There are also new female pelvis atlases which may improve bladder contouring.

DSCs greater than 0.5 were found when comparing AI-generated structures to the AP structure, with the exception of RAD femoral head owing to the contouring differences outlined above. Most structures had average DSC scores between 0.7 and 0.9, indicating good agreement in the bulk of the structure but with room for improvement, especially at the periphery. Doolan et al. investigated five autocontouring solutions, including RAD, using volumetric methods [26]. Their work found similar DSC scores when averaged across all volumes for the various contouring solutions. They also investigated the time savings and found that between 14 and 93 min could be saved based on the number and complexity of the contoured organs. The average HD and MDA were similar between the autocontouring solutions, with the exceptions noted above. 41 out of 48 structures had an average MDA < 5 mm.

When examining physician scores between contouring modalities, 11/16 (68.8%) of the manually generated approved physician contours had average scores ≤ 2.5. MIM showed slightly worse results, with 10/16 (62.5%) with average scores ≤ 2.5, while both RAD and SIE achieved better results, with 14/16 (87.5%) of contours receiving average scores ≤ 2.5. Bustos et al. compared one autocontouring solution to manually generated and atlas-based contours [27]. Their work also included a review of the AI-generated contours by a single radiation oncologist and found that of the 140 contours evaluated, only 5 (3.6%) required major edits or were completely redone. A total of 95 (67.9%) were judged to be clinically useable with no edits necessary, similar to the results of this study. We deemed contours with average physician scores less than 2.5 be clinically usable, with only minor or stylistic differences. With most of the AI-generated contours achieving these scores, all investigated products can be deemed to be at least as good as physician contours for a subset of contours. This underscores the potential of AI-generated contours to simplify and streamline the contouring and treatment planning process.

As a result of this work, it was decided to implement AutoContour (RAD) at all our clinical sites spanning five facilities, four CT simulators, eight LINACs and three HDR treatment units. Whilst similar physician scores and similarity metrics were found with all vendors, at the time of this work, RAD had the largest number of available organ contours.

## Conclusion

The results of our comparison demonstrated that each vendor’s AI contouring solution exhibited similar capabilities, with no striking differences in contouring accuracy or efficiency. The consistency and comparable performance of all three vendors’ solutions suggest that radiation oncology centers can confidently choose any of the evaluated solutions based on individual preferences, resource availability, and compatibility with their existing clinical workflows.

Notably, physician-generated contours received an average physician score of 2.02, which was worse than that of two of the AI contouring solutions, highlighting the variability among physicians in manual contouring and the potential of standardization that AI tools may offer. The accuracy of AI contouring is heavily reliant on the quality and diversity of the training data, as well as the robustness of the underlying deep learning algorithms. This is highlighted with examples of unusual anatomy presented and the corresponding poor physician scores and volumetric metrics. Although AI-based contouring may result in high-quality contours for most patients, a minority of patients require manual contouring and more in-depth physician review. Ensuring the adaptability of the AI model to diverse patient populations and anatomical variations remains a crucial challenge that demands further research and development.

The continued advancement of AI technologies in radiation oncology holds promising potential for further enhancing treatment planning precision and efficiency, especially with the increasing utilization of adaptive radiation therapy (ART). For ART, a patient’s treatment plan is modified over the course of treatment based on the observed changes in the tumor and surrounding normal tissues, which is an area of increased interest as departments strive to offer improved and individualized treatments to patients. As the field progresses, it is crucial for researchers, clinicians, and vendors to collaborate closely, continually refine, and validate AI contouring algorithms to ensure the highest level of clinical accuracy and patient care.

## Data Availability

No datasets were generated or analysed during the current study.

## Abbreviations

AI: Artificial intelligence
AP: Manually generated contours approved by the treating physician
DSC: Dice similarity coefficient
HD: Hausdorff distance
HU: Hounsfield unit
MDA: mean distance to agreement
MIM: MIM ProtégéAI+
OARs: organs at risk
RAD: Radformation AutoContour
SIE: Siemens DirectORGANS
